# The Lived Experience of Pediatric Pain Management: Insights From Families and Providers

**DOI:** 10.1177/23743735261455630

**Published:** 2026-05-27

**Authors:** Samrithaa Balakrishnan, Annesha Dey, Aarti Bavare, Paul Checchia, Katherine Shwetz, Natasha Afonso

**Affiliations:** 1 Medical Humanities Research Institute, 3990 Rice University, Houston, TX, USA; 2 Division of Critical Care, Department of Pediatrics, Baylor College of Medicine, 3984 Texas Children’s Hospital, Houston, TX, USA

**Keywords:** pediatric pain management, parental involvement, healthcare provider-family relationships, individualized care

## Abstract

Pain represents a subjective experience that manifests with emotional and physical discomfort. In pediatric care, pain management is especially complex due to communicative limitations, which creates a unique point of entry for parents to contribute to their child’s treatment process. In this study, we explored whether families’ perceptions of their child’s pain and preferences for treatment were aligned with healthcare providers’ pain management decisions. Using narrative-informed techniques in interviews with families and healthcare providers in pediatric care settings, we analyzed areas of concordance and discordance in pain assessment and treatment priorities. Our findings show that trust is built over time through (1) consistent communication and (2) individualized care for their child’s unique needs. Contrastingly, when trust is broken, families experience added stress with care. Future research should prioritize developing tools and interventions that support this collaborative model. Ultimately, these efforts aim to equalize power dynamics in the family-provider relationship by emphasizing the value of both parental understanding and medical knowledge in pediatric pain management.

## 1. Introduction

Pain can be defined as a subjective experience which encompasses the emotional and psychological implications of physical discomfort.^1^ The subjective nature of pain perception creates ambiguity in how we understand pain. Additionally, cultural beliefs play a significant role in shaping how individuals perceive and respond to pain, which can influence treatment outcomes.^2^ Self-reported pain is considered the gold standard of pain assessment.^3^ Specifically, combining both objective and subjective methods into multi-dimensional approaches to pain management can prove useful in measuring pain.^4^

Pediatric pain management presents unique challenges in assessing pain, due in part to developmental barriers; young children are limited in their communicative ability to use vocabulary and in their cognitive ability to articulate their pain.^5^ The distinctive challenges in pediatric pain management create a vital role for parents in recognizing and advocating for their child’s pain management.^6^ Due to the subjective nature of pain, parents may struggle to understand their child’s pain or communicate it effectively to the medical team.^7^ Recent literature of pediatric analgesic strategies emphasizes the need for individualized dosing and multimodal regimens.^8^ Factors such as disparities in theoretical knowledge, families’ spiritual or cultural beliefs, and immense pressure on both doctors and families create an environment fraught with tension and disagreement.^9,10^ Lastly, because children differ substantially in pharmacokinetics and pharmacodynamics, even identical analgesic dosages can yield markedly variable therapeutic and adverse responses.^11^ This variability places pediatric patients at risk of (1) undertreated pain, associated with impaired recovery, and (2) overmedication, which can provoke sedation, respiratory depression, and tolerance.^12^

Frequently used self-reported pain assessment scales (e.g. Faces Pain Scale-Revised (FPS-R) Face, Legs, Activity, Cry, Consolability (FLACC) scale) have been shown to help with pain assessment in pediatric patients. However, their utility as a point of parental entry is limited. For example, parents are often not provided with enough education about pain scales to warrant proper use in assessing the patient’s pain level.^13^ Additionally, many of these pain scales are limited to English-speaking patient families.^14^ Pain is an inherently subjective and biopsychosocial experience, and assessment of the meaning of pain demands a more comprehensive, individualized perspective than parents–or any outsider–can provide.^15^

In cases of chronic pediatric pain, parents are often responsible for advocating for their child’s pain management, an obligation which can lead to feelings of isolation and exhaustion.^16^ Given these challenges, we explore both parental and provider perspectives on the pediatric pain management process to gain a more comprehensive understanding of the alignment of family-provider perspectives.

## 2. Methods

This qualitative descriptive study began in January 2024, with the aim of exploring individual families’ reflective preferences regarding pain management strategies. Individual interviews were conducted with (1) the patient’s family (any members present during data collection), and (2) a member of the patient’s healthcare team (either attending physician or nurse, depending on availability during data collection). Our goal was to investigate whether families’ perceptions of their child’s pain and treatment preferences aligned with providers’ pain management decisions.

### 2.1. Setting and Participants

This study was set in the 48-bed cardiac intensive care unit (CICU) at Texas Children’s Hospital in Houston, Texas. As a quaternary referral pediatric hospital with over 950 beds, the facility receives patients from various hospitals and regions across the United States, which offers diversity in terms of communication styles and medical preferences of our sample. Convenience sampling methods were used to interview 31 families and corresponding healthcare teams over a four-month period. Cognizant of the intense emotional toll placed on families with critically-ill children, we sought patients whose families would be comfortable participating in reflective discussions of their child’s care. This did not correspond to selecting patient families with primarily-positive experiences or fewer conflicts with the healthcare team; rather, we selected patients that had the capacity and willingness to speak about their medical experiences. In selecting patients, we were guided by the charge nurse or attending physicians’ assessment of the family’s readiness/capacity for being interviewed. In this context, “readiness” referred to the clinical team’s assessment that the family was not in an acute medical crisis, had sufficient emotional bandwidth to participate in a 20 minute interview, and was not actively engaged in immediate procedural consent discussions. Despite using a convenience sampling method, we made a deliberate effort to ensure heterogeneity with a variety of cardiac conditions, language/translation preferences (English, Spanish, Arabic), and age range (from 1 month to 12 years old). While this approach prioritized ethical sensitivity in an emotionally intense environment, we acknowledge that it may have introduced selection bias by favoring families perceived as collaborative with the healthcare team. Families experiencing acute distress, conflict, or medical instability may therefore be underrepresented in our sample.

### 2.2. Interview Approach

**Figure 1.**
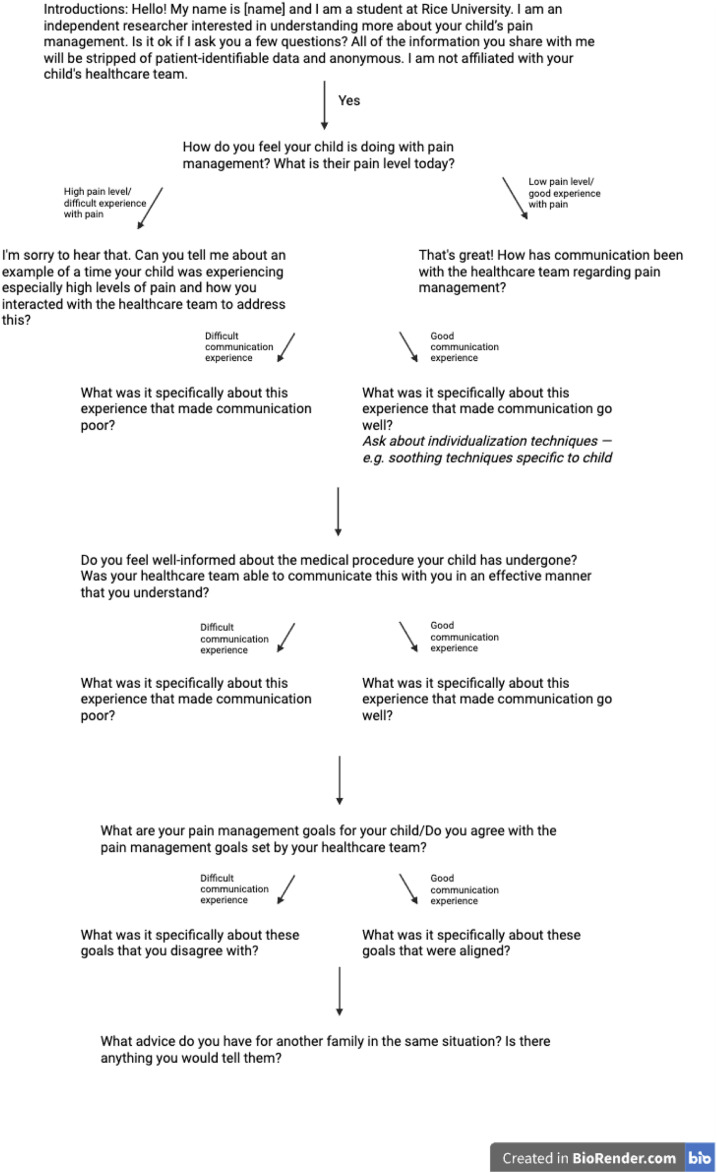
Semi-structured interview guide for exploring parental experiences with pediatric pain management, outlining the branching logic of questions posed to parents based on their child’s reported pain level and perceived communication quality with healthcare providers

Interviews were informed by a narrative-based approach to encourage reflection. Questions were open-ended, progressing from general prompts to more specific inquiries (see Figure 1). A small preliminary set of interviews [n = 10] informed refinement of the interview guide; all subsequent interviews were with different families. Providers were interviewed first to establish clinical background and rationale for management strategies. Families were then interviewed regarding their perceptions of pain management, communication, and roles in decision-making. Interviews ranged from 10-30 minutes, with an average of 20 minutes.

To minimize participant burden and encourage openness in this high-stress environment, we opted not to audio-record interviews.^17^ Instead, during each interview, one researcher asked questions while another took detailed field notes with verbatim phrases, affect, and gestures. These were expanded immediately afterward into transcribed field notes, with identifiers removed.^18^ One of our investigators (KS) assisted in refining the interview questions and implementing narrative-based interviewing techniques into the question list.

The research team included clinicians and scholars within the pediatric cardiac intensive care environment and academia. Our familiarity with either clinical culture or academia facilitated contextual understanding. However, we recognize that our institutional affiliation could have influenced how participants framed their experiences, particularly in a setting characterized by high emotional intensity and differences in power. Families may have perceived the interviewers to be aligned with the healthcare team, potentially influencing their willingness to share criticism or be vulnerable in the conversation. To mitigate this, all interviews began with a clear statement that the interviewers were not members of the child’s healthcare team and that participation would not influence the patient’s care. Participants were informed that their responses would remain confidential and would not be communicated back to the healthcare team.

### 2.3. Data Analysis

Written notes were interpreted using thematic analysis.^19^ Following their six-phase process, we: (1) familiarized ourselves with the notes by repeated reading; (2) generated initial codes inductively; (3) collated codes into categories; (4) reviewed and refined categories into candidate themes; (5) defined and named themes; and (6) produced the final thematic structure.

A coding-reliability approach was utilized to enhance credibility. Two researchers independently coded each interview, and interpretations were shared among the entire team of six until consensus was reached. Codes reflected such topics as parental entry points into care, trust and mistrust in providers, prior healthcare experiences, communication practices, and use of non-pharmacological strategies. We compared narrative-based descriptions of specific positive and negative interactions with families, noting qualitative expressions of affect. We maintained shared spreadsheets documenting coding decisions and evolving theme structures, providing transparency akin to an audit trail. The primary researchers (SB and AD) regularly consulted with the full team for insights on medical expertise (NA, AB, and PC) and medical humanities (KS) to refine their analysis. Although formal coding began after completion of all interviews, our research team informally reviewed field notes throughout the data collection period. As interviews progressed, we noted recurring narrative patterns across both provider and family accounts, with few substantively new insights emerging in later interviews. Recruitment concluded when our team reached consensus that additional interviews were unlikely to generate new conceptual understandings related to pain management. Saturation was therefore determined through a team-based discussion and iterative review of the full dataset.

## 3. Results

We conducted 62 interviews with patient families and healthcare (HC) teams, 31 with parents (including 6 non-English-speaking families) and 31 with providers (2 physicians, 29 nurses). We categorized our assessment of family-provider collaboration surrounding pain into three overarching themes: (1) communication, (2) individualized care, and (3) trust.

### 3.1. Communication

#### 3.1.1. Comments on Pain Management Collaborations & Parental Inclusion

##### 3.1.1.1. Healthcare Team Perspectives

Healthcare teams respected parental insight, prioritized ongoing education, and strove to create a collaborative environment. Nurses prioritized parental collaboration in pain management decisions, with one noting that “the pain management process is the only place where parents have control over the entire medical process.” Providers explained that parents have a generally good understanding of when their child is in pain; for example, one nurse caring for a non-verbal patient expressed that the mother’s understanding of the patient’s mannerisms was useful. Nurses additionally detailed strategies of parental collaboration, explaining that they work alongside parents to eliminate all possible causes of patient distress “as a team” before resorting to pain medications. For example, nurses establish a baseline of what “no pain” looks like with parents, asking them to report any changes or indications of medication tolerance. To ensure parents remain informed, nurses explained what to look for in observable signs and symptoms.

Several healthcare providers also characterized parental attendance during medical rounds as a form of parental involvement in pain management. They expressed that they encouraged parental involvement during rounds, specifically to create opportunities for parents to raise concerns regarding weaning processes. Additionally, nurses shared that debriefed rounds with parents afterwards helped address pain and general medical concerns, such as asking “can I re-explain anything for you?”

##### 3.1.1.2. Parental Perspectives

Parents reported the healthcare team’s efforts at inclusion: one parent noticed the healthcare team checking for the signs of pain she had described (e.g. chewing tongue, smacking lips), and another described the pain management process as a “mix of what parents know and what nurses have found.” Indeed, one parent mentioned that she regularly worked with the healthcare team to figure out which consoling techniques would work because her child responded to different strategies on a daily basis. Parents also expressed that rounds were helpful for understanding the pain management process, and shared that they felt comfortable asking questions and raising concerns during rounds.

#### 3.1.2. Parental Perspectives — Language Barriers to Understanding and Communication

Six out of the thirty-one families interviewed had a non-English language preference (NELP), which led to distinctive challenges. Some NELP families reported challenges when an in-person translator was unavailable, citing long wait times for online translators and frequent technological issues, such as video interruptions. Additionally, a lack of awareness about translator services left some NELP families frustrated and unable to fully understand medical rounds. Families in situations where no same-language-speaking providers were present noted they were informed of interventions only after they had been implemented, rather than receiving step-by-step explanations during the process. This led many families to express a preference for having a same-language-speaking provider on the care team. Other NELP families reported no concerns with translator services and felt that communication with the care team was generally effective.

#### 3.1.3 Healthcare Team Perspectives — Communication Strategies

The healthcare team reported the use of several strategies to ensure clear communication, depending on each family’s situation. For families unable to be present at the hospital for extended periods, they provided a summary of everything that occurred in the family’s absence and ensured they were available to answer questions when the family was present. Other strategies include asking about the patient’s pain levels in the morning. When translator services were needed, providers emphasized the importance of keeping the family informed about medications and interventions. Another strategy involved directly engaging the patient in conversations about their pain.

#### 3.1.4 Parental Perspectives — Changing Medical Teams

Families reported communication challenges stemming from frequent changes in medical teams, both during day/night shift transitions and weekly rotations. For one family, this resulted in repeated attempts at pain control strategies that had previously proven ineffective. Additionally, frustrations arose with repeatedly explaining the patient’s situation to new medical teams and uncertainty about whom to approach when parents needed to voice concerns. Other parents shared that communication has generally been good, stating that they were well informed about what to expect despite the rotating teams.

### 3.2. Individualized Care

#### 3.2.1. Healthcare Team Check-Ins & Addressing Concerns

##### 3.2.1.1. Healthcare Team Perspectives

Healthcare providers noted that they always tried to be available for parents to raise concerns. Strategies mentioned include specifically asking about pain levels in the morning and giving “rundowns” when parents haven’t been in the hospital for extended periods, bringing in a physician where necessary. One nurse expressed that she felt parental concerns were well-addressed by physicians during rounds, especially those regarding additional administrations of pain medications. Others mentioned ensuring a social worker was called when parents expressed financial concerns.

##### 3.2.1.2. Parental Perspectives

Several parents shared that nurses frequently checked in for parental concerns and to assess their child’s pain levels. One parent expressed that they “didn’t feel like a bother,” with others describing the nurses as “going above and beyond” and “very attentive.” Contrastingly, one parent expressed that check-in frequency depended on the nurse, with “some nurses being more present than others.”

Parents generally, but not unanimously, felt that their questions and concerns were sufficiently addressed. For instance, some parents expressed worries about transitioning out of the cardiac ICU or requested specific physical therapy scheduling, both of which were addressed. One parent noted that her child was distressed by frequent physical contact, and the healthcare team responded by sedating the patient to reduce stress. Another parent raised a skin concern, which a nurse alleviated by examining the skin under a light and explaining her findings. In contrast, two parents expressed that their concerns about potential overmedication were dismissed and not adequately addressed.

#### 3.2.2 Consoling Strategies

##### 3.2.2.1. Healthcare Team Perspectives

Healthcare providers reported being attentive to parental preferences for non-pharmacological comfort measures, including swaddling or cradling the infant, gently rubbing the infant’s back, or adjusting the bed for pediatric patients, before considering pharmaceutical interventions. In some cases, providers adopted consoling methods they observed parents using. Strategies also included eliminating all other reasons for the patient to be crying (e.g. checking diapers) before administering medication. Additionally, multiple pain scales were used to assess pain before giving additional doses of pain medication or only administering medication if the patient was “inconsolable.”

##### 3.2.2.2. Parental Perspectives

Some parents expressed a preference for utilizing consoling techniques prior to administering medication, explaining that they preferred “simpler” methods over “harsher” medications. They shared that the healthcare team understood and respected their preferences, with one saying that she “can rest easily knowing they will try soothing techniques before drugs.” A few parents expressed that they appreciated the balance between pharmaceuticals and non-clinical soothing techniques. One parent attributed this variability to differences in nursing engagement, stating that it “depends on the nursing team that day.”

### 3.3. Establishment or Breakdown of Trust

In this collaboration, trust emerges as both a goal and a barometer. Thus, we posit that these two standards—consistent communication and individualized care—ultimately culminate in either fostering or reducing trust in the medical team. The former improves overall satisfaction with care while the latter intensifies families’ already-traumatic clinical experiences in a damaging way.

Parents were more likely to accept a healthcare provider’s suggestions, even if initially skeptical, when a foundation of trust had been established. For instance, even though some parents expressed they did not agree with the amount of medication their child received, those that had developed strong trust relationships with the healthcare team were able to nonetheless accept clinical guidance, including the necessity and risks of untreated pain.

Some parents specifically expressed that they “tried to put trust” in the medical team’s decisions. This task was often easier after witnessing consistent communication and value for parental input and concerns, and was more stressful after trust had been previously broken.

In relationships characterized by comparatively weaker trust, parents were less willing to accept clinical recommendations. For example, one parent explained an incident in which she felt that her child had been administered too many medications. She detailed her experience, explaining that she “would’ve stopped it if [she] understood, but [she] felt very helpless and out of the loop.” She shared that she “felt as though [she] let [her] child down as a mother,” articulating a sense of responsibility and guilt. This left families feeling scared and helpless when decisions were not clearly communicated in advance.

Some families expressed a preference for physicians to address the emotions surrounding the medical decision rather than focusing solely on the clinical aspects. One mother shared that her doctor was “more on the phone making decisions” than “spending time talking to [her] about her feelings about everything.” When asked what advice she would give to other families in a similar situation, this parent shared that it’s important to be your child’s advocate, “even when you’re exhausted.” Negative healthcare experiences led this parent to have a severe lack of trust in the healthcare system, causing her to feel she has to stay awake to the point of exhaustion to ensure her child remains stable.

In contrast, parents who experienced more significant communication with their healthcare team felt increased trust and comfort in their decisions. For example, a parent who had flown to receive care specifically at this institution expressed that he initially felt very stressed when he didn’t know what was going on, highlighting that this was caused by unclear communication in previous medical experiences at other institutions. However, he noted the effort the medical team put into ensuring his complete understanding of his daughter’s conditions, even showing him how to operate the morphine pump. Over time, he shared that he grew to trust that the medical team’s decisions were “always” in his child’s best interest. Given that we have conceptualized pain management as a point of entry for parents into their child’s care, this exemplifies a meaningful opportunity for parents to actively participate in alleviating their child’s pain. “I understand now that every cord is for good reason,” he shared.

## 4. Discussion

While prior literature has emphasized that parents provide a unique perspective on their young child’s pain experience,^20^ this study further explores how these perspectives align with those of providers in real-time. By simultaneously comparing provider and family narratives, our findings reveal that points of discordance in pain management emerge from breakdowns in the relationship. Though trust and communication have been established as essential components of the family-provider relationship,^21^ this study’s narrative-informed, qualitative methodology contributes a novel view of specific factors that build trust through a relational, ongoing process.

Rather than presenting these challenges as problems with clear-cut solutions, this study aims to emphasize the gray areas that define much of pain management. Each interaction observed between families and the medical team was shaped by an evolving blend of clinical expertise, emotional context, and situational pressures. Thus, pain management involves navigating uncertainties with compassion and adaptability, emphasizing clear communication and transparent partnership with families.

### 4.1. Communication

Beyond facilitating strong collaborations between families and providers, communication functioned as a central mechanism through which families evaluated care quality. This aligns with the concept of interaction quality, which refers to how care is delivered through interpersonal processes rather than solely what care is delivered. Even when clinical management was technically appropriate, inconsistent or inaccessible communication, particularly among families requiring interpreters, diminished perceived quality of care. Thus, the quality of communication that accompanies clinical outcomes is itself an important determinant of how families perceive care.

Clinically, these insights point to several actionable strategies in the pediatric intensive care setting. For example, documenting family narratives of their hospital experience can reduce the burden on families to repeatedly explain their medical history and care preferences to changing medical teams. Second, efforts should be made to prioritize language concordance between nurses and families and to ensure availability of translation services; bilingual nurses expressed that communicating directly with families facilitated care. Ultimately, families and providers should make every effort to engage in conversations that value each others’ perspectives.

### 4.2. Attentive Individually-Tailored Care

Individualized care operates as an extension of shared decision-making, where parental expertise is treated as clinically actionable knowledge. For instance, when the parent defines a concern in care, which might include new symptoms or changes in insurance coverage, the clinician changes the plan of care to reflect their lived reality.^22^ This principle informs actionable additions that can be incorporated into care. For example, nurses can proactively check in and invite parents to share their pain management strategies. These preferences, such as for consolation, can be documented along with a family’s general narrative to ensure continuity in individualized pain management approaches across changing medical teams.

### 4.3. Holistic Outcomes of Lack of Trust vs Trust

Trust arises as a downstream outcome of communication quality and inclusion in decision-making in pediatric pain management. Mistrust often occurs when healthcare providers fail to adequately include families in pain assessment, leading to parental feelings of exclusion,^23^ which can give rise to parental anxiety and mistrust, introducing further complications into pain management efforts.^24^ Language barriers increase the likelihood of misinterpretation, leading to exacerbation of trust deficits as parents feel misunderstood or marginalized in clinical settings.^25^ Additionally, strong relationships that are founded on effective communication enable providers to learn from parental expertise. This allows the relationship between family and provider to extend beyond symptom management, giving the provider a deeper understanding of the unique needs of both the family and the child.

An essential component of pediatric clinical practice is trauma-informed care. When providers engage in behaviors that cultivate trust within the family-provider relationship, they help buffer families against the stress and potential retraumatization associated with hospitalization. Conversely, when relational trust is disrupted, the clinical encounter itself may intensify distress and heighten the risk of retraumatization.^26^

## 5. Limitations & Future Directions

While this study provides important insights into pain management in pediatric intensive care settings, it is important to acknowledge its limitations. Since it was a single-site study, the generalizability of our findings to other institutions is limited. Additionally, the use of convenience sampling methods might have introduced selection bias, favoring families who were more comfortable discussing their experiences. Since data was collected through interviews that relied on retrospective self-reporting, recollections might have been influenced by emotional states, recall bias, or social desirability bias. In addition, the study’s manner of data collection did not allow for longitudinal tracking of families, limiting our ability to understand how perceptions of pain management evolve over time. In the future, efforts should be made to track the same families throughout their hospital stay to examine how their relationship with the healthcare team evolves over time.

Future work should translate these findings into targeted interventions and quality improvement efforts, or policy changes within pediatric intensive care settings, such as:

1. Language equity strategies: Additional investment into interpretation infrastructure in multilingual and diverse care environments.
2. Family engagement practices: Physicians can incorporate 1-2 narrative-based questions into their rounds to further invite parents to share their concerns, preferences, and holistic hospital experience.
3. Increased family advocacy: Family liaisons or patient advocates can be included in decision-making to facilitate communication and relieve parental burden for advocacy.

## 6. Conclusion

Our study confirmed the complexities involved in assessing and collaboratively managing pediatric pain. A key finding that arose is the role of consistent communication, individualized care, and their role in the establishment or breakdown of the trust-based relationship between families and providers. As we move toward obtaining a better understanding of pediatric pain management, it is essential to acknowledge the nuanced realities providers and families face where trust, communication, and individualized care serve not as simple fixes, but as essential tools for navigating the gray areas that define clinical practice.
